# Anxiety and depression affect pain drawings in cervical degenerative disc disease

**DOI:** 10.1080/03009734.2017.1319441

**Published:** 2017-05-15

**Authors:** Anna MacDowall, Yohan Robinson, Martin Skeppholm, Claes Olerud

**Affiliations:** a Department of Surgical Sciences, Uppsala University Hospital, Uppsala, Sweden;; b Department for Learning, Informatics, Management and Ethics, Karolinska Institutet, Stockholm, Sweden

**Keywords:** Cervical spine, degenerative disc disease, Hospital Anxiety and Depression Scale, pain drawings, pain modality, surgical treatment

## Abstract

**Introduction:**

Pain drawings have been frequently used in the preoperative evaluation of spine patients. Until now most investigations have focused on low back pain patients, even though pain drawings are used in neck pain patients as well. The aims of this study were to investigate the pain drawing and its association to preoperative demographics, psychological impairment, and pain intensity.

**Methods:**

We carried out a *post hoc* analysis of a randomized controlled trial, comparing cervical disc replacement to fusion for radiculopathy related to degenerative disc disease. Preoperatively the patients completed a pain drawing, the Hospital Anxiety and Depression Scale (HADS), and a visual analogue scale (VAS). The pain drawing was evaluated according to four established methods, now modified for cervical conditions. Comparisons were made between the pain drawing and age, sex, smoking, and employment status as well as HADS and VAS.

**Results:**

Included were 151 patients, mean age of 47 years, female/male: 78/73. Pain drawing results were not affected by age, sex, smoking, and employment status. Patients with non-neurogenic pain drawings according to the modified method by Ransford had higher points on HADS-anxiety, HADS-depression, and HADS-total. Patients with markings in the head region had higher score on HADS-depression. Markings in the neck and lower arm region were associated with high values of VAS-neck and VAS-arm.

**Conclusions:**

Pain drawings were affected by both pain intensity and anxiety/depression in cervical spine patients. Therefore, the pain drawing can be a useful tool when interpreting the patients’ pain in correlation to psychological impairment and pain location.

## Introduction

Pain drawings have been a common tool, allowing patients to communicate pain without the necessity of an elaborate language for quite some time. As early as 1949 Palmer (1) wrote about pain drawings and how to distinguish between functional and organic pain. Since then pain drawings have been analysed in order to see if we in fact can draw any conclusions about the different ways patients fill in the pain drawings. Several methods of assessing the pain drawings have been developed, and these assessment methods have been compared to psychological scales (2–11), radiology methods (12–16), and treatment outcome (17–25).

Until now most investigations have focused on low back pain patients, even though pain drawings are frequently used in neck pain patients as well. Cervical radiculopathy is caused by degenerative changes such as disc herniation or foraminal narrowing due to decreased disc height, pleated ligament, and osteophyte formation of the uncovertebral and/or facet joints. The most commonly affected nerve root is the C7, secondly the C6. The symptoms are neck pain with arm pain in the same distribution area as the affected nerve (26). To our knowledge, a thorough study of the role of pain drawings in preoperative assessment for cervical degenerative disc disease (DDD) has not been done.

This study was designed to evaluate whether pain drawings of neck pain patients are affected by: (1) age, sex, smoking, and employment status; (2) anxiety and depression; and (3) pain intensity.

## Patients and methods

This study was a *post hoc* analysis of a prospective randomized controlled trial (RCT) of 151 patients from three hospitals in Sweden during 2007 through 2010. The patients suffered from radiculopathy due to DDD and were randomized after exposure and decompression to either artificial disc replacement, ADR (Discover™, DePuy Spine, Johnson & Johnson, Raynham, MA), or plated fusion using autologous iliac crest graft. Inclusion and exclusion criteria as well as two-year results have been published previously (27).

On the day before surgery the patients completed a questionnaire with demographic details, a pain drawing, the Hospital Anxiety and Depression Scale (HADS), and a visual analogue scale (VAS) (Table 1).

**Table 1.** Demographics at baseline.

Patient characteristics	Total *n*	
Age, years, median (min–max)	130	46 (31–61)
Women/men, *n*	130	67/63
Smokers, *n* (%)	130	39 (30)
Non-smokers, *n* (%)	130	91 (70)
In work, *n* (%)	128	111 (87)
Not in work, *n* (%)	128	17 (13)
High-HADS, *n* (%)	129	39 (30)
Low-HADS, *n* (%)	129	90 (70)
High-VAS-neck, *n* (%)	127	55 (43)
Low-VAS-neck, *n* (%)	127	72 (57)
High-VAS-arm, *n* (%)	128	48 (38)
Low-VAS-arm, *n* (%)	128	80 (63)

High-HADS ≥10 points; Low-HADS <10 points; High-VAS ≥67; Low-VAS <67.

The study was approved by the Regional Ethical Review Committee in Stockholm (Dnr: 2006/1266-31/3). Patient informed consent was obtained before randomization. The study was registered at ISRCTN (registration number: 44347115).

### Pain drawing

The pain drawing developed by Spangfort (28), which is a modified version from Ransford et al. (2), was used. The test consists of a front and back outline drawing of the human body. The patients indicate the distribution and the character of their present pain using six different symbols: dull, burning, numbness, stabbing or cutting, pins and needles, and cramping (Appendix 1). Three spinal surgeons scored the pain drawings independently. For evaluation of the pain drawings we used the penalty point system by Ransford et al. (2), the visual inspection method by Udén et al. (13), the grid assessment method by Gatchel et al. (29), and scoring by body surfaces by Ohnmeiss (12). The evaluation methods have been validated for the cervical spine (unpublished observation).

### Penalty point system by Ransford

The pain drawing was assigned points for following characteristics: unreal drawings (indications of pain in patterns inconsistent with radicular symptoms), drawings showing ‘expansion’ or ‘magnification’ of pain (indicating pain outside the drawing of the body), ‘I particularly hurt here’ indicators (using arrows or extra words to emphasize pain intensity), ‘Look how bad I am’ indicators (a tendency to demonstrate total body pain). A score of two points or less was regarded as normal (Appendix 2). The penalty point system by Ransford was modified to the cervical spine and is henceforth referred to as the modified Ransford method.

### Visual inspection method by Udén

The visual inspection method by Udén was modified to the cervical spine as follows:

- Neurogenic (N)—the pain drawing shows pain in the arm and/or shoulder as in typical nerve root pain.
- Possible neurogenic (PN)—the pain drawing shows some aberrations from a classic nerve root syndrome.
- Non-neurogenic (NN)—the pain has a distribution that could not be explained by radiculopathy.
- Possible non-neurogenic (PNN)—the pain drawing shows very little resemblance with a nerve root pain and is therefore hard to categorize into the other groups above.

The visual inspection method by Udén is henceforth referred to as the modified Udén method.

### Grid assessment method by Gatchel

The pain drawing was divided by bilaterally symmetrical grids with small boxes of approximately equal area. The grid over the human figure was copied onto a transparent plastic template and placed over each completed pain drawing for scoring. The number of boxes filled in by markings was counted.

### Scoring by body surfaces by Ohnmeiss

The method was modified for cervical use; hence the pain drawing was divided into the following five regions: neck, head, upper trunk (scapula region), upper arm, and lower arm. Markings on the elbow or wrist non-contiguous with neck or arm pain were disregarded because they may indicate joint problems (12). We used a transparent plastic template with the human figure containing the boundaries placed over each completed pain drawing for scoring (Appendix 1). The scoring by body surfaces by Ohnmeiss is henceforth referred to as the modified Ohnmeiss method.

### Anxiety and depression

The HADS (30) is a 14-item instrument where seven questions concern anxiety (HADS-a) and seven concern depression (HADS-d). Every item scores on a four-point scale from 0 to 3, resulting in a maximum score of 42. It is a validated tool in medical practice for screening psychological distress in non-psychiatric patient populations (31,32). Falavigna et al. have presented a cut-off for HADS-d ≥ 10 points with a sensitivity of 71.1% and a specificity of 95.4% for patients undergoing spine surgery (33).

### Pain

The VAS consisted of a 100-mm horizontal line with the description ‘no pain’ on the far left and ‘worst possible pain’ on the far right (34). The patients were asked to make a vertical mark on the line to show the location that best represented the pain they had experienced during the last week. The patients received separate VAS for neck and arm pain.

### Statistics

The modified Ransford and Udén methods were dichotomized to neurogenic/non-neurogenic according to the original articles. The Gatchel method was dichotomized according to Takata and Hirotani (Table 2) (22). HADS was dichotomized into one group with high values (high-HADS) if either anxiety or depression score was equal or more than 10 points, and one group with low values (low-HADS), with an anxiety and depression score <10 points, based on Falavigna et al. (33,35). VAS was divided into three groups where the highest third, 67–100, was classified as high-VAS. The lower third, together with the medium values, was classified as low-VAS.

**Table 2.** Principles of dichotomization of the various methods.

Method	Neurogenic	Non-neurogenic
Ransford (penalty points)	0–2	3+
Udén	N, PN	NN, PNN
Gatchel (ticked boxes, *n*)	0–19	20+

### Predictor analysis

For the dichotomous method that refers to N/NN (i.e. dichotomized versions of modified Ransford, Udén, and Gatchel methods), we examined whether a selected set of baseline variables predict a NN outcome. To avoid over-fitted models, only four predictors were used: age, sex, smoking, and employment status.

A logistic regression model was fitted with NN as outcome, and the four mentioned predictors as independent variables. Since there were three independent observers there are three values per patient. Hence for the ‘total’ observer, the observations were dependent. Therefore, a random effects logistic regression was run in this case, with the patient as a random intercept. From these models, odds ratios (OR), confidence intervals (CI), and *P* values were extracted for each predictor.

In the modified Ohnmeiss method there was an imbalance with a small number of patients in one group out of two in every region. For instance, 97% of the patients had marked pain in the lower arm region, and only 3% had not. Such a distribution makes logistic regression with four different variables unreliable, and it was therefore not carried out.

### Validation

The following values were correlated to the pain drawings: HADS-total (HADS-t), HADS-a, HADS-d, high-HADS, low-HADS, high-VAS, low-VAS. The inferential part was done only for the dichotomous/dichotomized pain drawing methods. For each such method and observer (including a ‘total’ one, pooling the results from all three observers), the endpoint values for the N and NN groups were compared. The endpoint mean for the N group was subtracted from the endpoint mean in the NN group. For the modified Ohnmeiss method, the comparison was instead between groups 0 (no pain markings) and 1 (with pain markings) separately for each body surface region. The endpoint mean for the group 0 was subtracted from the endpoint mean in group 1. Positive values correspond to larger values for the NN group or, for modified Ohnmeiss, for group 1.

The target parameter was the difference in means. Confidence intervals and *P* values were computed using bootstrap with *B* = 10,000 bootstrap replicates and the percentile method (36). *P* values of <0.05 were considered significant. For the ‘total’ observer, we resampled patients (i.e. triplets of values) rather than individual values, reflecting the dependence between values for the same patient.

Finally, some dichotomous endpoints were compared to dichotomized pain drawing results. The endpoints in question were dichotomized HADS and the two (dichotomized) VAS values. This comparison was made using the Fisher exact test. The odds for having a NN pain drawing was computed for each dichotomized group, and then the OR was calculated, which expresses the probability of having a NN pain drawing between the two HADS or the two VAS groups. The numerator was the odds for high-HADS and high-VAS, respectively, which means that OR >1 favours the association between the NN pain drawings and high-HADS, high-VAS. We also computed confidence intervals and *P* values for the null hypothesis of no association between NN pain drawings and HADS groups and no association between NN pain drawings and VAS groups (OR = 1).

Missing data were handled using ‘available cases’. Hence only patients without missing values for the variables used in the analysis at hand were included. Consequently, the populations the various analyses were based on are not the same. No correction was done for multiple testing/estimation. All statistical analyses were performed in *R* (37), version 3.1.0 (2014-04-10), x86_64-w64.

## Results

Of the 151 patients included in the RCT, 20 patients had missing data for pain drawings. One pain drawing was incorrectly given after the operation. Two patients were lacking employment status, one patient was lacking HADS, three patients were lacking VAS-neck, and two patients were lacking VAS-arm.

None of the chosen preoperative demographic factors (age, sex, smoking, and employment status) were related to a NN pain drawing in any of the three assessment methods (Table 3).

**Table 3.** Results from predictor analysis. Entries are odds ratio, OR (95% CI) *P.*

Method	Age: years****OR (95% CI) *P*	Gender: male versus female****OR (95% CI) *P*	Smoking****OR (95% CI) *P*	Employment****OR (95% CI) *P*
Ransford	0.99 (0.94, 1.04) 0.62	1.30 (0.65, 2.60) 0.46	1.97 (0.92, 4.23) 0.81	0.99 (0.36, 2.75) 0.99
Udén	0.98 (0.91, 1.06) 0.63	1.68 (0.61, 4.57) 0.31	2.57 (0.88, 7.47) 0.084	0.56 (0.14, 2.24) 0.41
Gatchel	0.83 (0.66, 1.06) 0.14	0.41 (0.01, 12.53) 0.61	24.29 (0.65, 903.88) 0.084	0.33 (0.00, 25.08) 0.62

The HADS-t value was lower in all N groups (median, 10 points) and higher in all NN groups (median, 13 points) independently of which method had been used, modified Ransford, modified Udén, or Gatchel. The HADS-t value was also lower if there were markings in the upper arm, but the value was higher if there were markings in the region of the head, neck, and lower arm (Table 4).

**Table 4.** HADS per method result for the totality of all observers. Entries are median (min, max).

Method	Mean no. of patients	HADS-d Median (min, max)	HADS-a Median (min, max)	HADS-t Median (min, max)
Ransford				
Neurogenic	64.5	4 (0, 13)	6 (0,16)	10 (0, 29)
Non-neurogenic	65.5	5 (0, 16)	7 (1, 16)	13 (1, 29)
Udén				
Neurogenic	93.75	4 (0, 16)	7 (0, 16)	10 (0, 29)
Non-neurogenic	36.25	5 (0, 16)	7 (1, 16)	13 (1, 29)
Gatchel				
Neurogenic	58	4 (0, 13)	6 (0, 16)	10 (0, 29)
Non-neurogenic	72	5 (0, 16)	7 (0, 16)	13 (1, 29)
Ohnmeiss				
Head				
0	101.75	4 (0, 13)	7 (0, 16)	10 (0, 29)
1	28.25	7 (0, 16)	7 (1, 16)	15 (1, 29)
Neck				
0	26	4 (0, 12)	7 (1, 16)	10 (1, 27)
1	104	5 (0, 16)	7 (0, 16)	12 (0, 29)
Shoulder				
0	6	4 (0, 16)	8 (2, 13)	10 (2, 29)
1	124	4 (0, 16)	7 (0, 16)	11 (0, 29)
Upper arm				
0	12	4 (0, 12)	7 (1, 15)	13 (2, 27)
1	118	4 (0, 16)	7 (0, 16)	11 (0, 29)
Lower arm				
0	4.25	2 (0, 9)	5 (2, 14)	6 (2, 23)
1	125.75	4 (0, 16)	7 (0, 16)	12 (0, 29)
Bilateral markings				
0	98.25	4 (0, 16)	7 (0, 16)	11 (0, 29)
1	31.75	5 (0, 13)	7 (1, 16)	10 (1, 27)

Patients with NN pain drawings, according to the modified Ransford method, had higher points on HADS-d (OR, 1.0; 95% CI, 0.1 to 1.9), HADS-a (OR, 1.0; 95% CI, 0.0 to 2.0), and HADS-t (OR, 2.0; 95% CI, 0.3 to 3.8). Patients who had made markings in the head region, when the modified Ohnmeiss method was applied, had higher score on HADS-d (OR, 1.7; 95% CI, 0.1 to 3.2) (Table 5). These findings were also supported by the analysis with the dichotomized HADS. There were 39 patients in this study group (30%) with either a HADS-a or a HADS-d value of 10 points or more. There was a higher risk for patients with high-HADS also to have a NN pain drawing according to the modified Ransford method (OR, 1.68; 95% CI, 1.06 to 2.66). High-HADS was also associated with markings in the head region on the pain drawing (OR, 2.34; 95% CI, 1.36 to 4.03) (Table 6).

**Table 5.** Results from method comparison. Entries are difference in means (95% CI) *P* value. The values are: The mean difference between HADS-d in group N and NN, group 1 and 0; The mean difference between HADS-a in group N and group NN, group 1 and group 0; The mean difference between HADS-t in group N and NN, group 1 and 0.

Method	HADS-d Mean diff. (95% CI) *P*	HADS-a Mean diff. (95% CI) *P*	HADS-t Mean diff. (95% CI) *P*
Ransford	1.0 (0.1, 1.9) 0.030	1.0 (0.0, 2.0) 0.046	2.0 (0.3, 3.8) 0.021
Udén	0.5 (–0.4, 1.4) 0.26	0.1 (–1.0, 1.2) 0.86	0.6 (–1.2, 2.5) 0.52
Gatchel	1.1 (–0.1, 2.2) 0.064	0.9 (–0.4, 2.2) 0.19	1.9 (–0.3, 4.1) 0.088
Ohnmeiss			
Head	1.7 (0.1, 3.2) 0.037	1.2 (–0.6, 3.0) 0.17	2.9 (–0.1, 5.9) 0.062
Neck	0.9 (–0.5, 2.3) 0.20	0.0 (–1.7, 1.7) 0.96	0.9 (–1.8, 3.6) 0.49
Shoulder	0.1 (–4.3, 3.4) 0.97	–0.3 (–2.9, 2.7) 0.84	–0.2 (–6.9, 5.8) 0.95
Upper arm	0.6 (–1.5, 2.4) 0.54	–0.4 (–2.7, 1.9) 0.74	0.2 (–3.9, 4.0) 0.91
Lower arm	1.7 (–2.2, 4.6) 0.33	0.5 (–6.2, 4.4) 0.83	2.3 (–8.4, 8.5) 0.61
Bilateral	0.2 (–1.2, 1.5) 0.82	0.1 (–1.6, 1.9) 0.90	0.3 (–2.6, 3.1) 0.85

Difference in means presented for each method. The endpoint mean for the N group is subtracted from the endpoint mean in the NN group. For the modified Ohnmeiss method, the endpoint mean for the group 0 (no markings) was subtracted from the endpoint mean in group 1 (with markings). Hence positive values correspond to larger values on HADS for the NN group or (for Ohnmeiss) for group 1.

**Table 6.** Analysis of dichotomized HADS and dichotomized preoperative VAS-neck and VAS-arm. The numerator was the odds for high-HADS and high-VAS, respectively, which means that OR > 1 favours the associations between the NN pain drawings (for the modified Ohnmeiss method, group 1, with markings) and high-HADS, high-VAS.

Method	HADS OR (95% CI) *P*	VAS-neck OR (95% CI) *P*	VAS-arm OR (95% CI) *P*
Ransford	1.68 (1.06, 2.66) 0.026	1.13 (0.74, 1.73) 0.60	0.84 (0.54, 1.29) 0.46
Udén	0.92 (0.54, 1.54) 0.80	0.90 (0.56, 1.46) 0.73	0.91 (0.55, 1.48) 0.72
Gatchel	1.21 (0.76, 1.92) 0.44	1.32 (0.86, 2.02) 0.21	1.46 (0.94, 2.27) 0.091
Ohnmeiss			
Head	2.34 (1.36, 4.03) 0.001	1.45 (0.86, 2.46) 0.16	0.67 (0.38, 1.17) 0.15
Neck	0.92 (0.52, 1.67) 0.78	2.56 (1.41, 4.79) < 0.001	0.53 (0.31, 0.90) 0.017
Shoulder	1.13 (0.37, 4.16) 1.00	0.75 (0.26, 2.20) 0.63	1.21 (0.41, 4.02) 0.81
Upper arm	1.03 (0.47, 2.39) 1.00	0.79 (0.38, 1.65) 0.49	1.28 (0.59, 2.89) 0.59
Lower arm	0.97 (0.27, 4.42) 1.00	2.62 (0.66, 15.03) 0.16	∞ (1.89, ∞) 0.003
Bilateral	1.01 (0.58, 1.73) 1.00	0.77 (0.46, 1.28) 0.33	0.93 (0.55, 1.56) 0.81

CI = confidence interval; HADS = Hospital Anxiety and Depression Scale; OR = odds ratio; *P* = *P* value; VAS = visual analogue scale.

Pain drawings analysed with the modified Ohnmeiss method were associated with both VAS-neck and VAS-arm. There were 55 patients (43%) with a VAS-neck value higher than 66 of 100. Most of the patients who marked pain in the neck region on the pain drawing were also in the high-VAS-neck group (OR, 2.56; 95% CI, 1.41 to 4.79). Forty-eight patients (38%) had a VAS-arm value of more than 66 of 100. All of them had marked pain in the lower arm region (OR, ∞; 95% CI, 1.89 to ∞). There was no association between high-VAS-arm values and markings in the upper arm region (Table 6).

## Discussion

This study documents for the first time the effect of anxiety and depression on cervical pain drawings. Findings of ‘non-organic’ pain drawings in low back pain patients have been associated with ethnic background, health insurance (13), female patients, previous spine surgery, and being unemployed (25). Interestingly for cervical spine patients, age, sex, smoking, and employment status were not associated with non-neurogenic pain drawings. Hägg et al. (17) and McNeill et al. (24) have presented similar findings.

Only few studies relate pain drawings to anxiety and depression (8,9,38). The HADS values in our study were very low in general, in both the neurogenic and non-neurogenic groups, implying that surgeon selection was a possible reason for the HADS values being lower than anticipated. Still, there were 39 patients with a value of 10 points or more in either anxiety or depression score.

In this study, four different assessment methods for pain drawings were applied, all of which had different characteristics. The method by Ransford emphasizes arrows, circled areas, explanatory notes, as well as generalized pain and strange pain patterns. In our study those features seemed to have a correlation to higher HADS values. The method by Udén disregards Ransford’s concepts (‘magnification of pain’, ‘I particularly hurt here indicators’, and ‘Look how bad I am indicators’) and focuses only on the pain patterns/‘unreal drawings’. In our study these features were not related to anxiety, depression, or pain. As a third option, the method by Gatchel disregards pain patterns/unreal drawings as well as ‘I particularly hurt here indicators’ and highlights only how widespread the markings, how generalized the pain is. Half of the patients (55%) had more than 19 boxes filled with markings according to the Gatchel method, e.g. a tendency towards generalized pain, but those patients did not have high-HADS values (≥10 points) or high-VAS values (>66). One feature unique to the method by Ransford is the inclusion of ‘I particularly hurt here indicators’, e.g. the arrows, explanation marks, circled areas. Since these were associated with more anxiety and depression, as measured with HADS, the greater sensitivity of the modified Ransford method to HADS was probably due to this particular feature.

The modified Ohnmeiss method focuses on the exact location of the patient’s pain. Markings in the head region were associated with high HADS values and markings in the neck and lower arm region with high values on VAS-neck and VAS-arm, respectively. The modified Ohnmeiss method predicts even surgical treatment outcome for cervical spine patients (unpublished observation).

There is no consensus about the interpretation of pain drawings. There are articles with and without associations when similar comparisons were made. Cultural differences may influence the results, Swedish studies being more consistent in reporting correlations between pain drawings and psychological impairment (3,17) as well as discriminating between neurogenic and non-neurogenic pain (39). Since other studies from countries with different health care and insurance systems arrive at the same conclusion as the Swedish studies (2,4,6,7), individual differences may be equally common within the same country (11) as between different cultures (9). From this perspective, it seems to matter more which region is investigated, the lumbar spine or the cervical spine (38,40), and if the patient has radiculopathy or not (12,13,15,40,41).

This analysis of anxiety and depression on pain drawings is relevant since there have been recent reports of high values on HADS being a negative predictor of surgical treatment outcome in cervical spine patients (35). Based on this study, we suggest the pain drawing as a possible first assessment-screening instrument in helping clinicians to select patients who might need further psychological screening. Compared to other patient-reported measurements and questionnaires, a pain drawing is very simple and quick, easy for the patient to understand, and also cheap. It is an important complement in the communication with the patient as not everyone communicates well with words and a pain drawing can then be very helpful. We therefore hope for more future research on this topic.

### Limitations

One major limitation of this study is the homogeneous study population due to the clear inclusion criteria in the RCT this study was based on. Therefore, one should be cautious in generalizing these results to other diagnoses.

The statistical calculations were done without correction for multiple testing/estimation. We hereby accept the risk of making a type one error. Since we did not compute so many variables, we estimated that with a correction for multiple testing the results would be too conservative, hence at risk of a type two error. According to false discovery rate, we had only approximately 1% risk of making a type one error, which we appraised to be a small risk (42).

## Conclusions

Age, sex, smoking, and employment status did not predict markings on the pain drawing whether they are neurogenic or non-neurogenic in patients with cervical DDD with radiculopathy. Pain drawings assessed as non-neurogenic, according to the modified Ransford method, were associated with anxiety and depression. Pain markings in the head region on the pain drawing were associated with depression. Markings in the neck and arm region on the pain drawing had excellent correlation to high values on VAS-neck and VAS-arm, respectively. We therefore suggest the introduction of pain drawings also for the preoperative assessment of cervical spine patients.

## Appendix 2

### The modified Ransford method. Hypothesis for evaluating pain drawings in the cervical spine

**Hypothesis for Evaluating Pain Drawings in the Cervical Spine**

Patient nb: _____________  □ pre. op  □ 3 m (4w)  □ 2 y (1y)

A patient with poor psychometrics may show this by:

1. *Unreal drawings* (poor anatomic localization, scores 2 unless indicated; bilateral pain not weighed unless indicated)
  - total arm pain
  - lateral whole arm pain (tuberculum majus area and lateral upper arm allowed)
  - circumferential upper arm pain
  - bilateral anterior under arm pain (unilateral allowed)
  - circumferential hand pain (scores 1)
  - bilateral hand pain (scores 1)
  - use of all four modalities suggested in instructions ( we feel patient is unlikely to have ”burning areas,” stabbing pain, pins and needles, and numbness all together
    SCORE: ______
2. *Drawings showing* ”expansion” or ”magnification” of pain (may also represent unrelated symptomatology; bilateral pain not weighted)
  - neck pain radiating to head, shoulder, thoracic or lumbar spine (each scores 1; scapula pain allowed)
  - elbow pain
  - wrist pain
  - pain drawn outside the outline; this is particularly good indication magnification (scores 1 or 2 depending on extent)
    SCORE: ______
3. ”*I Particularly Hurt Here*” indicators
  Some patients needing to make sure the physician is fully aware of the extend of symptoms may: (each category scores 1; multiple use of each category is not weighted)
  - add explanatory notes
  - circle painful areas
  - draw lines to demarcate painful areas
  - use arrows
  - go to excessive trouble and detail in demostrating the pain areas (using the symbols suggested)
    SCORE: ______
4. ”*Look How Bad I Am*” indicators
  Additional painful areas in the trunk, head, lumbar spine or lower extremities drawn in. Tendency towards total body pain (scores 1 if limited to small areas, otherwise scores 2).
  SCORE: ______

TOTAL: _______  □ normal (score > 2)  □ suggests poor psychometrics
